# Policy instruments as a trigger for urban sprawl deceleration: monitoring the stability and transformations of green areas

**DOI:** 10.1038/s41598-024-52637-9

**Published:** 2024-02-01

**Authors:** Krisztina Filepné Kovács, Dalma Varga, Anita Kukulska-Kozieł, Katarzyna Cegielska, Tomasz Noszczyk, Milan Husar, Vera Iváncsics, Vladimir Ondrejicka, István Valánszki

**Affiliations:** 1 Department of Landscape Planning and Regional Development, Hungarian University of Agriculture and Life Sciences, Villányi Street 29-43, 1118 Budapest, Hungary; 2 Department of Landscape Protection and Reclamation, Hungarian University of Agriculture and Life Sciences, Villányi Street 29-43, 1118 Budapest, Hungary; 3 Department of Land Management and Landscape Architecture, Faculty of Environmental Engineering and Land Surveying, University of Agriculture in Krakow, 21 Mickiewicza Avenue, 31-120 Krakow, Poland; 4 Spectra Centre of Excellence of the EU, Slovak University of Technology in Bratislava, Vazovova Street 5, 812 43 Bratislava, Slovakia

**Keywords:** Ecosystem services, Urban ecology

## Abstract

The socialist era postponed suburbanisation in Central and Eastern European countries. After 1990, the process became extremely intensive and transformed the compact form of cities into more decentralised and dispersed urban structures. Therefore, the study aims to identify the main trends in land-cover transformation caused by urban sprawl in peri-urban areas of three Central and Eastern European cities (NUTS 3 level: the Pest County, Bratislava Region, and Krakowski subregion). In addition, we identified various policy tools for green infrastructure protection. We further investigated the extent to which the presence of legal means of nature conservation affects the stability of natural and seminatural areas. The research used an original questionnaire and spatio-temporal analysis. It has been confirmed that after decades of socialism, a highly intensive urban sprawl process started in the analysed regions. It generally slowed down after 2000 except for the Krakowski subregion. The majority of new artificial areas replaced agricultural land. Despite the dynamic urban sprawl, almost one-third of the analysed Central and Eastern European peri-urban areas were stable natural and seminatural areas. The traditional nature conservation tools proved to be effective in preserving natural and seminatural areas, but the protection of landscapes exposed to urban sprawl needs specific tools. The effectiveness of urban sprawl control is hindered by the fact that spatial planning competences are dispersed. This research may influence monitoring urban sprawl and offer an innovative method because it combines spatial analysis (quantitative approach) with the impact of policy tools (qualitative approach).

## Introduction

### General overview of urban sprawl

Cities have come to the forefront of attention; they play a crucial role as engines of the economy. Their impact extends far beyond their administrative boundaries and affects their surrounding areas. One of the commonly tackled phenomena relevant to this issue—urban sprawl—consists of all the means of expansion of urban living. The concept appears in several disciplines, and a growing number of studies deal with its various aspects^1,2^. These interact with the social, infrastructural, and regulatory environment of cities^3,4^. The investigation of the topic is particularly exciting in post-socialist countries the history of which has spatial effects to this day^5,6^.

Compared to Western Europe, Central and Eastern European countries exhibit a lower level of urbanisation in general. Yet, a significant increase in artificial surfaces took place in the region, especially in urban areas after 1990^7–9^. Many studies have addressed the differences between urbanisation patterns found in Western and Central and Eastern Europe. Most of the studies highlighted as a key characteristic that the socialist era postponed or interrupted the process of suburbanisation. However, it became extremely intensive and transformed the compact form of the cities into a more decentralised and dispersed urban structure after 1990^10^.

According to Schuchman^11^, the main reasons for suburbanisation in Central and Eastern Europe—slightly different than in Western Europe—were: the physical and social degradation of inner residential areas, the lack and delay of rehabilitation, the functional transformation of the central city after 1990, gentrification, migration of worse-off classes out of the downtown. Furthermore, the need for residential areas changed generally in society and the Western type of living and consumption model grew more popular.

Based on Eurostat^12^ data for Central and Eastern European countries, the ratio of artificial areas was still below the EU average in 2018, which is 4.2% of the total area—HU: 4.0%, SK: 3.4%, PL: 3.6%—mostly far below Western-European countries (DE: 7.6%, BE: 11.7%, AT: 4.4%; FR: 5.7%).

The stability of landscape, especially the stability of natural and seminatural ecosystems, is on the opposite side of the spectrum to the growth in artificial areas. The stability can be manifested in complex patterns with local changes, but on a larger scale, it remains the same shape^13^. On the landscape level, stability refers to the spatial and functional stability of land-use categories in time^14^. Generally, it is the share of stable areas in the analysed period, which can give a general overview of the socio-economic processes and natural features^15^.

Urban expansion provokes conflicts all over the world, including in developing countries through the degradation of ecosystems^16^, changes in landscape patterns, increasing landscape fragmentation^17,18^ or loss of cropland^19^. Changes in natural areas, especially green spaces, caused by the sprawling of cities are common challenges faced by many countries, in particular developing ones. Koprowska et al.^20^ explored the link between urban sprawl and the availability of urban green spaces in Łódź (Poland) in this regard. Similarly, Sperandelli et al.^21^ tried to understand the relationship between urban sprawl and the spatial development of green and vacant land in the Metropolitan Fringe of Sao Paulo (Brazil).

However, green spaces and agricultural land close to large cities are declining in many world regions^22^. Such a trend is observed in China^23^, Europe^24^, and Africa^25^. Rapid urban expansion profoundly affects global biodiversity through habitat conversion, degradation, fragmentation, and species extinction^26^. Therefore, natural and seminatural areas are particularly important in peri-urban areas, where sprawl is the main type of development^27,28^.

### Policy instruments for protecting green infrastructure in peri-urban areas

Green infrastructure is a strategically planned network of natural and seminatural spaces. It is often discussed in the literature in relation to urban growth and planning, especially in the context of the loss of green spaces and farmland in peripheral areas^25^. According to Howlett^29^, policy instruments are specific measures of implementing policy objectives that are available to governments. There are many diverse classifications of policy instruments. Nevertheless, the most common ones—which we also used during our research—are: (1) regulatory instruments (directly control specific aspects related to certain spaces), (2) economic instruments (related to economically-oriented approaches), and (3) informational and motivational instruments (awareness building and educating social actors)^30,31^. In agglomeration areas, the instruments need to be combined to improve the efficiency of governance and decision-making^32^. According to European documents, this could be a mixture of different legislation, guidelines, programmes, as well as structural funds, which is referred to as a ‘policy mix’^30^.

Regulatory instruments (1) mostly as tools of spatial planning constitute the largest group. Agglomeration areas related to our research topic exhibit several types of these instruments, such as: (a) limits on urban growth, strict regulations regarding construction control, and greenbelt planning as a specific tool (e.g. Swiss Federal sectorial plan for crop rotation areas); (b) nature and landscape protection (e.g. Natura 2000 system in Europe); (c) green infrastructure planning, designation of regional ecological corridors, and greenway planning as a specific tool (e.g. the greenbelt and greenway system of Rennes Metropole in France)^33^.

The economic instruments (2) are mainly specific compensation tools for the loss of ecological values and for development. These activities (e.g. construction of roads and other development) are considered necessary for the public. However, compensation is often required for lost ecological value. In Switzerland, ecological compensations are compulsory for large construction projects^34^, in Germany the so-called eco-accounts (*Ökokonto*) intends to address the impact of developments^35^.

Informational and motivational instruments (3) are the most flexible and voluntary tools. Apart from awareness building and educating social actors, this group also includes the intermunicipal cooperation on comprehensive planning. Cities need to look beyond their borders and cooperate with the municipalities in their functional area^33,36^.

### Aim and novelty of the paper

The study aims to identify the main trends in land cover transformation caused by urban sprawl in Central and Eastern European peri-urban areas. In addition, we investigated the extent to which the presence of legal means of nature conservation affects the stability of natural and seminatural areas.

To this end, we posed the following research questions:

- Are green infrastructure protection tools applied in Central-European peri-urban areas and what are they?
- What are the most important unique and common trends in urban sprawl in the study area?
- What is the spatial distribution of stable natural and seminatural areas in the study area? How are these areas located relative to core cities?

The novelty of the research is the preparation of an original questionnaire to evaluate policy tools for urban sprawl control and the protection of green infrastructure. The questionnaire is highly versatile as it can be applied to any other research area. Furthermore, the study represents a new perspective; it combines different research methods from social sciences and engineering. This way, we (1) analyse the legal aspects affecting urban sprawl control and protection of green infrastructure and (2) identify and map the spatial distribution of stable land cover and the transformation of land cover due to urban sprawl.

## Materials and methods

### Study areas

**Figure 1:**
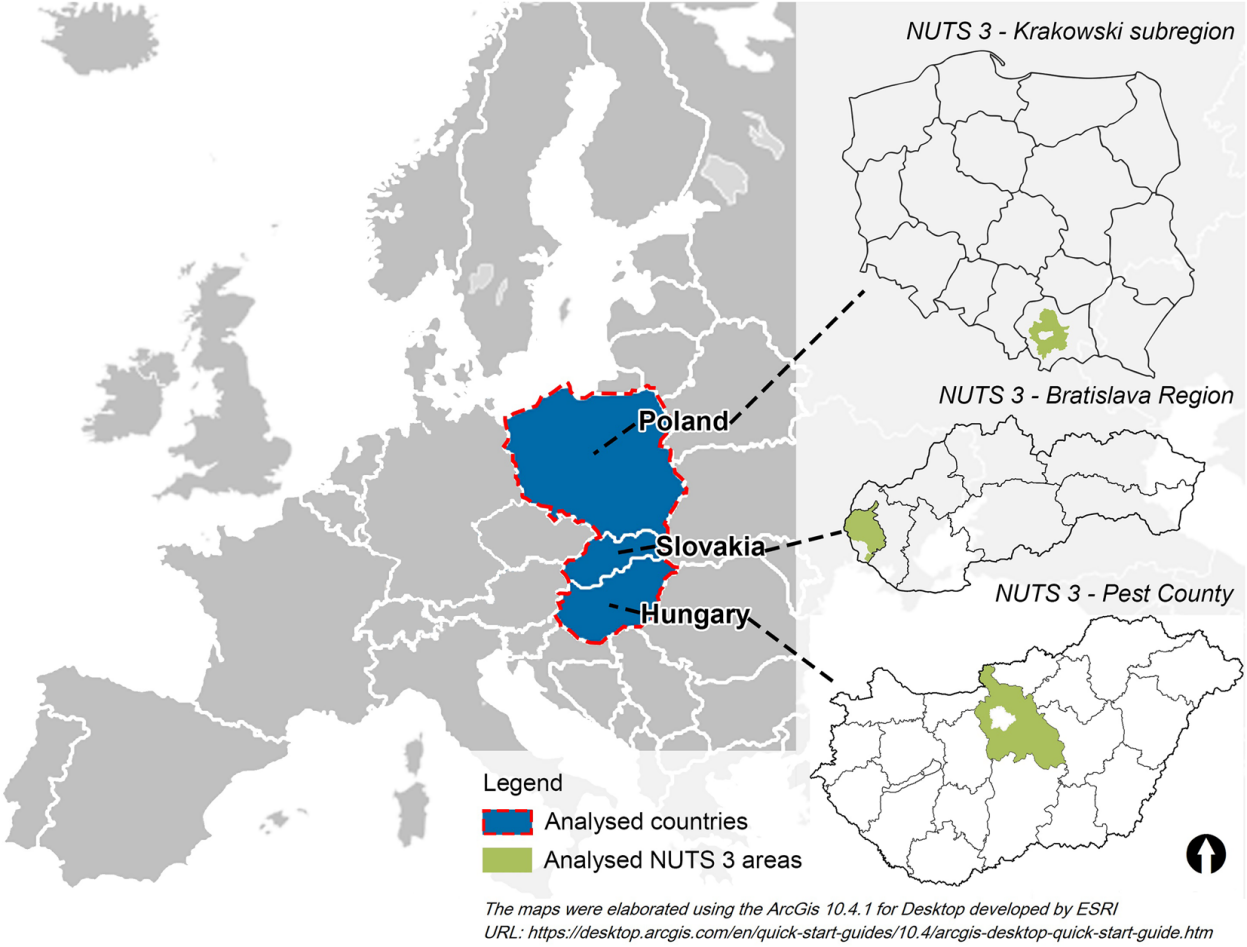
The NUTS 3 study areas.

The area chosen for the study is Central and Eastern European peri-urban areas from three countries: Hungary, Slovakia and Poland (Fig. 1). As urban agglomeration zones are defined and delimited differently in these countries, we adopted the European NUTS nomenclature to unify the research areas. Accordingly, the study involves three NUTS 3 units: the Pest County (Hungary), Bratislava Region (Slovakia), and Krakowski subregion (Poland).

The Hungarian study area—Pest County—covers 18 districts (187 municipalities). This area and the capital city are home to 30% of the country's population. Suburbanisation started intensively after 1990: Budapest lost 250,000 inhabitants in two decades. The area has diverse landscapes. An agglomeration zone was defined around Budapest containing 81 settlements for which a land use plan was adopted as an act of law in response to the intensive suburbanization and concentration processes after 1990.

The Slovak area investigated here—Bratislava Region—contains eight NUTS4 regions (five of which form the city of Bratislava) and 73 municipalities. Bratislava Region is the most efficient and most attractive region regarding migration and economy in the Slovak Republic^37^. The region is a varied geographical area and has a unique location. It neighbours two states at the same time—Austria and Hungary—creating a highly developed cross-border region called the ‘golden triangle’^38^.

The Krakowski subregion covers six districts (67 municipalities) and is inhabited by a total of over 720,000 people. The region's population has grown by more than 100,000 over the past three decades. The northern part of this region is dominated by agricultural land. The central and southern parts are the most urbanised. The most worrying phenomenon, according to Podhalański and Arvay-Podhalańska^39^, is the drive to develop natural ecological corridors, including those along river beds in the metropolitan area.

### Methods

The research procedure involves three methods. The first is desk research. It includes an analysis of the literature on the urban sprawl phenomenon specific Central and Eastern European agglomeration zones and policy tools or instruments. The second one is an original questionnaire. With this questionnaire, we identified what kind of urban sprawl control and green infrastructure protection tools are applied in Central and Eastern European peri-urban zones. The last method is the spatio-temporal analysis of land cover changes, with which we identified spatial land use changes (especially green infrastructure) between 1990 and 2018, as well as stable natural and seminatural areas.

#### Review of policy tools

Urban sprawl does influence land cover changes in peri-urban areas. It can, therefore, be assumed that the occurrence of this phenomenon does not provide adequate protection for the areas identified in the study as stable. We aimed to investigate whether the study areas analysed had tools that prevent urban sprawl and thus preserve the stability of natural and seminatural areas. The review was elaborated by the experts of the analysed countries to identify the most important policy tools for urban sprawl control and green infrastructure protection. We compared spatial strategies and policy tools for peri-urban areas (without the core cities), looking for how urban sprawl is controlled and environmental values protected.

The review contained three groups of questions (see Table A1 in Appendix). The first group explored spatial planning tools, such as general spatial planning tools, special actions to combat urban sprawl, and green infrastructure planning and protection tools. Considering the issue of the legal protection of nature, we highlighted differences in levels of protection and tools for the protection of green infrastructure, ecological networks/corridors, and greenway planning as a tool. As the third group of questions, we explored specific economic instruments for fostering compact settlement structure and

#### Spatio-temporal analyses

For spatio-temporal analysis, we used a methodologically coherent database commonly used and accepted in the European community covering a long interval for the whole study area: the Corine Land Cover (CLC) with data points for 1990, 2000, 2006, 2012, and 2018. Due to the universality of the CLC, its data provide ample opportunity to compare results between EU countries. The geometric accuracy for 1990 was ≤ 50 m, while in each subsequent year, the value improved, reaching ≤ 25 m and then ≤ 10 m in 2018^40^. Moreover, the CLC database provides vector data, unlike, for example, the GCL-FC30D database^41–43^. Although the latter provides data for the entire world, it is a raster database with a lower accuracy (30 m).

To ensure a general overview of the trends, we aggregated the CLC nomenclature, creating three main types of land cover: artificial, agricultural and natural, and seminatural land cover types (see Table A3 in Appendix). Following the methodology of Ronchi et al.^44^ and Skokanová et al.^45^, green infrastructure was assigned to natural and seminatural surfaces. We analysed the changes in the five periods. All the analyses, geographical locations of the objects, and visualisations were completed with ArcGIS 10.4.1 using GIS tools and geospatial analyses. We developed dedicated computation algorithms and employed advanced GIS research and analytical tools, primarily the Cartesian product, relational joins, and many more, including functions for computing statistical parameters. We also employed the vector overlay method to explore the growth of artificial surfaces. We then determined conversions from natural and seminatural areas and from agricultural areas to artificial areas in the investigated periods (1990–2000, 2000–2006, 2006–2012, 2012–2018) using the algebra of vector maps and SQL query.

As the second step, we explored the stability of natural and seminatural areas. We accepted as stable those areas whose use type remained unchanged from 1990 to 2018. To determine which natural and seminatural areas were stable, map algebra was used again. In the next step, the structure of nature protection means and their share in stable natural and seminatural areas was examined (Appendix A2). The following indicators were calculated:

1 $${{\varvec{I}}}_{{\varvec{P}}{\varvec{x}}}=\boldsymbol{ }\frac{\sum_{{\varvec{i}}=1}^{{\varvec{n}}}{{\varvec{P}}}_{{\varvec{x}}{\varvec{i}}}}{{{\varvec{A}}}_{{\varvec{j}}}}\times 100\boldsymbol{\%}$$

where I_Px_ is the ratio of xth nature protection means to the area of the jth NUTS3; P_xi_ is the area of the ith part of land covered by the xth nature protection means (i = 1, …, n); A_j_ is the area of the jth NUTS3.

Therefore, I_Px_ indicates which part of the NUTS 3 area is legally protected.

2 $${IP}_{xSNS}= \frac{\sum_{k=1}^{m}{P}_{xkSNS}}{\sum_{i=1}^{n}{P}_{xi}}\times 100\%$$

where IP_xSNS_ is the ratio of stable natural and seminatural areas covered by the xth nature protection means to the total area of the xth nature protection means in the investigated unit (such as the jth NUTS 3). P_xkSNS_ is the area of the kth part of a stable natural and seminatural land covered by the xth nature protection means (k = 1, …, m). IP_xSNS_ indicates which part of the nature protection means type remained stable over the investigated period.

3 $${ISNS}_{Px}= \frac{\sum_{k=1}^{m}{P}_{xkSNS}}{\sum_{l=1}^{r}{SNS}_{l}}\times 100\%$$

where ISNS_Px_ is the ratio of stable natural and seminatural areas covered by the xth nature protection means to the total area of stable natural and seminatural land cover types in the investigated unit (such as the jth NUTS 3). SNS_l_ is the area of the lth part of the stable natural and seminatural area (l = 1, …, r).

The last indicator PSNS_Px_ shows what part of stable natural and seminatural areas is covered by a given nature protection means. Labels (x) of means of nature protection are used in Appendix A2 as well.

Natura 2000 sites were analysed separately, as it is a European-level nature protection tool, designated based on universal guidelines. In addition, aggregated values for selected protected areas were calculated for each NUTS 3 area. We noted the national-level protection areas in individual countries in the study area, such as in Hungary: the national park, the landscape protection area, the nature protection area; in Poland the national park, the protected landscape area, the natural preserve, the landscape park; in Slovakia: the protected landscape area, the small protected area. There is some kind of protection of the ecological network in all countries.

## Results

### Tools for urban sprawl control and green infrastructure protection

#### Spatial planning instruments

The first aspect analysed in the questionnaire concerned spatial planning policy instruments (Table 1). In addition to nature protection tools, spatial planning is the most important tool to protect ‘everyday’ landscapes. Hence, the authors first explored how the institutional framework functions and what kind of tools are there for peri-urban areas in the investigated countries.

**Table 1:** Spatial planning tools.

	Pest county	Bratislava region	Krakowski subregion
Institutional background regarding spatial planning (regional level)	On the NUTS 3 level, the county council is responsible but has weak planning competencies. It does not have authority over the Budapest agglomeration zone There is a state secretary responsible for Budapest and agglomeration development	On the NUTS 3 level, there are legal entities in Slovakia (Bratislava Region), but with weak planning responsibilities	There is no common institution responsible for spatial planning on the NUTS3 level. Planning is institutionalised on the NUTS 2 level Municipalities are the most important level of spatial planning
Intermunicipal cooperation to foster common planning	There is no planning association/institution on the agglomeration level, just a state secretary Weak responsibilities on the NUTS 3 level No other intermunicipal cooperation on the relevant regional level	There is no common institution for agglomeration planning in Bratislava Weak responsibilities on the NUTS 3 level No other intermunicipal cooperation on the relevant regional level	There is no planning association/institution or relevant inter-municipal cooperation No other intermunicipal cooperation on the relevant regional level
Public participation tools	It is required by the law to inform the public in the process of drafting local plans, but mostly it is not necessarily a real involvement	It is required by the law to involve the public. However, in practice, it usually stays on the level of informing, possibly consultation	It is required by the law to allow the public to share their opinion on draft masterplans and local spatial development plans
Tools for controlling/preventing urban sprawl	The county land use plan does not have direct tools, but the land use plan of the Budapest agglomeration zone has regulations to control sprawl	No tools to control urban sprawl directly. The masterplan clearly states the limits of development and development areas	Only at the local level—regulations in local spatial development plans concerning development indicators
Tools for protecting unbuilt areas	The strategic objective is to protect agricultural and forest land based on sectoral acts National- and regional-level land use plans have rules for specific land use types, e.g. forest or high-quality agricultural land The strongest tool on the local level is the balancing of built-up areas in land use plans	The strategic objective is to protect agricultural and forest land based on sectoral acts Only local-level balancing of built-up areas in masterplans. Protection of the primary function of the land and protection of high-quality arable land	The strategic objective is to protect agricultural and forest land based on sectoral acts Tools only on the local level—balancing of built-up areas in masterplans
Tools for protecting green infrastructure	Green infrastructure is not protected as such. It is only included in land use plans from the national to local level as part of the ecological network. Furthermore, certain regulatory zones, such as the zone of forest, also provide protection	Green infrastructure is integrated into spatial plans under obligatory regulations It is included in spatial plans from the national to local level as part of the Terrestrial System of Ecological Stability	Only on the local level—regulations in of local spatial development plans concerning the coverage of green areas
Ecological network in spatial planning	Act on Nature Conservation (No. LIII of 1996.) designates National Ecological Network areas. They are integrated into land use plans on the national level. Limited construction in the core and corridor zones, but a low level of protection for the buffer zone	Act No. 543/2002 on Nature and Landscape Protection designates the Terrestrial System of Ecological Stability, containing biocenters, biocorridors, and interacting elements of supra-regional, regional, or local importance	Act of 16 April 2004 on nature protection defines the concept of the ecological corridor. Despite the lack of direct references to ecological corridors in legal regulations related to spatial planning, this issue is covered at all planning levels to a varying extent, but especially in local spatial development plans
Tools for protected areas	From the national- to local-level land use plans, as part of the National Ecological Network. Nature protection areas, usually as core areas, are designated in land use plans. Construction activities are limited and a corridor area allows building just with a permit. On the local level, nature protection areas are taken into account	From the national to local level as part of the Terrestrial System of Ecological Stability. Different types of protected areas have their own designation and regulations that masterplans and investment projects need to consider. These are mostly limitations on changes related to functional use On the local level, protected areas are taken into account	On the local level protected areas are taken into account in local spatial development plans Also, on the regional level (NUTS 2), a system of protected areas is indicated in the regional spatial development plan. In addition, a landscape audit must be carried out on this level at least once every 20 years
Tools for compensation of loss of ecological values due to development	On the local level: a government decree prescribes the maintenance of the biological activity level in the administrative area of the settlement	The greenery index is specified for each area in the masterplan. For land grabs, authorities can prescribe what measures are to be taken to compensate for any biodiversity loss. For other forms of land development, these issues are not defined	The minimum percentage of the biologically active area is specified in the local spatial development plan for built-up areas. For other forms of land development, these issues are not defined

There is no common institution for agglomeration planning in all the analysed countries. Planning and cooperation mostly happen on the level of administrative units as is evident in Slovakia and Poland. Even though there is a master plan for Bratislava Region, nearly all planning decisions are made on the local level, and any major planning decisions are taken on the national level. In Poland, planning is institutionalised on the NUTS 2 and municipality levels (LAU 1). The Budapest region differs from the others. There is an agglomeration zone defined for its area with a land use plan, but common development planning is not supported. There is no common regional institution for the agglomeration zone just a representative of the state oversees the development issues.

The research demonstrated that despite the strong suburbanization in the study area^46–48^, no specific tools are applied for controlling sprawl. The only exception is Hungary, where a land use plan is drafted and adopted as an act of law for the agglomeration zone of Budapest. It provides for specific regulations to curb urban sprawl, such as limiting the growth to not more than 2% of the existing urban area and preventing the merging of urban areas. In Slovakia, there are no direct tools for controlling urban sprawl. The regional-level masterplan provides for development restrictions. As the most important level in spatial planning, local spatial plans are generally binding legal acts. Poland is the only country among the three, where local spatial development plans are not obligatory planning acts. On the local level, the spatial policy is implemented with two primary planning documents: the masterplan and the local spatial development plan, which is the basic instrument for spatial planning and an act of local law. In their absence, spatial management is affected by administrative decisions. Only the master plan is mandatory, but it is not an act of local law. Its regulations may differ from the building permits issued by the authorities.

The investigated countries usually have tools to protect unbuilt areas in local-level spatial plans (land use plans in Hungary, spatial plans in Slovakia, and local spatial development plans in Poland) with the obligation to maintain the primary function of land and protect farmland especially high-quality farmland (HU, SK, PL) or forests (HU, PL) and unbuilt areas with high natural value (all countries)^49^. In Slovakia and Hungary, the authorities also have several tools to protect agricultural land through various legal regulations related to land ownership, land use, land planning, and land protection^50,51^. In Poland, a one-off fee is paid for this as well as annual fees paid over 10 years^52^. Furthermore, master plans in Poland must take into account the real need for new investment areas from 2016. This ensures that unbuilt land is protected from an unreasonable change of use^53^.

The ecological network is a regulated part of spatial plans (Slovakia) and their equivalent, land use plans in Hungary. In Poland, on the other hand, there is no direct reference to taking ecological corridors into account in the Act on spatial planning and development. Despite this, the process of creating a spatial development plan (or another planning document) in Poland takes into account the identification of potential elements of the ecological network and can shape it through the appropriate provisions of the plan regarding the use and development of these elements. The investigated countries have tools for assessing the ecological value of the area of the municipality in local-level planning (biological activity value, greenery index etc.) to compensate for the loss of biodiversity. However, some practical problems are not uncommon, general guidelines fail in the practice (Slovakia). In Hungary, special governmental decrees can provide exemptions from the general rules.

Public participation is usually low in the analysed countries. However, it is mandatory to involve the public during the whole planning process^54^. In practice, it rarely goes beyond informing or possibly consultation. The problem is also connected with the expectations from both sides regarding the participation process. The public is usually unaware of the possibility and expresses their concerns at late stages. These findings are consistent with the study by Kaczmarek and Wójcicki^55^ who investigated the Polish city of Poznań.

#### Legal protection and other instruments

Another aspect analysed in the questionnaire was legal protection and economic instruments. We verified whether the countries have institutions responsible for the protection of green spaces and ecologically important areas. The means and tools for protecting these areas in the study area were also checked. We then looked into specific economic incentives (Table 2).

**Table 2:** Legal protection and economic tools.

		Pest county	Bratislava region	Krakowski subregion
Legal protection of nature	The institution responsible for the protection of green/ecologically valuable areas	Ministry of Agriculture On the regional level: county Environment and Nature Protection Authority, National Park Directorates	Ministry of Environment National Park Administrations, territorial units of Slovak National Forest Service	There is a high dispersion from the central to the local level, including specially established administrative bodies (e.g. Regional Directorate for Environmental Protection)
	National-level nature protection	1 National park 2 Landscape protection areas	2 protected landscape areas Several smaller nature protection areas	1 National park 6 Landscape parks Buffer zones that are protection zones around three means of nature protection (national parks, natural preserves, and landscape parks)
	International level protection	32 Natura 2000 sites 2 Ramsar sites	17 Natura 2000 sites 4 Ramsar sites	41 Natura 2000 sites
Economic instruments	Economic incentives for fostering compact settlement	VAT reduction when buying a new home in an area declared a rust belt	No	No
	Economic incentives for green infrastructure protection	No	No	Several city-level initiatives, such as tax exemption for a green roof or rainwater collection system subsidies
	Economic instrument—additional costs for the increase in property value	Levy, a zoning change fee when selling a property	No	Rezoning fee (a zoning change fee), betterment levy

Legal means of nature protection in the investigated areas are designated to protect the stability of green infrastructure. The legislator has introduced many means of nature protection, often managed by various institutions/authorities on national and regional level. This makes it difficult to uniformly manage and protect green infrastructure throughout the NUTS 3 as there are many independent responsible bodies sharing responsibility. In Hungary regrettably, it is a strong trend that economic interests outweigh environmental issues for the central government. Building and environmental decisions are taken away from the local level.

The three regions have several types of traditional national-level means of nature protection—from the national park to smaller nature protection areas with more or less overlapping Natura 2000 sites. There are no specific greenbelt regulations in the analysed regions.

Other instruments, such as economic, informational, and motivational tools can also foster more rational landscape management. The investigated countries have introduced some incentives (however not many, and mostly focusing on urban areas) to foster the protection of unbuilt areas and the preservation of green spaces. Hungary introduced a new policy tool to stimulate brownfield development, with a VAT reduction. Polish cities have incentives for green infrastructure solutions but mostly for built up areas. There are also incentives in the form of rainwater collection system subsidies or tax relief for improving the thermal performance of buildings. There are no specific financial tools to preserve and develop green infrastructure in Slovakia.

In Hungary and Slovakia, there are no effective economic instruments to recover the costs of public projects from property owners if their property value has increased due to public development projects. However, there have been discussions about the need for introducing such tools. In Poland, one of the economic instruments for recovering costs in connection with an increase in property valu**e** is the rezoning fee (a zoning change fee)^56,57^ and a similar instrument has been in place in Hungary since 2020. It is a one-off payment paid by the owner to the municipality if the value of their property has increased as a result of the adoption or amendment of a local spatial development plan, provided that the owner sells the property within five years from the day the local plan or its amendment became effective^56^. The seller is liable to pay a levy if they sell the property and its status had changed due to rezoning in the last 10 years. Another example from Poland is the betterment levy^58^.

### Stability and transformation of land cover

#### Loss of green infrastructure as a consequence of urban sprawl

The results on land cover changes highlight the transformation of green infrastructure elements and the conversion of agricultural areas and natural and seminatural areas into artificial areas. This confirms urban sprawl in the study area. This may also be related to the lack of tools for controlling/preventing urban sprawl identified in section "Tools for urban sprawl control and green infrastructure protection". Table 3 reflects the different trends and dynamics of the loss of natural and seminatural areas. In Pest County and Bratislava Region, the sum of agricultural and seminatural areas transformed into artificial type constituted 2.86% and 3.2% of the total area, respectively. In the Krakowski subregion, it exceeded 8% in the same timeframe. In the Slovak and Hungarian NUTS 3, approx. 90% of losses were in agricultural areas, while in the Krakowski subregion, agricultural land constituted almost 99% of the lost cover.

**Table 3:** Land cover conversion into artificial land cover type.

		1990–2000	2000–2006	2006–2012	2012–2018	Total
HU pest county	From seminatural and natural (km^2^)	7.01	2.21	5.62	2.3	17.14
	From seminatural and natural (%)	0.11	0.03	0.09	0.04	0.27
	From agricultural (km^2^)	100.73	22.65	35.17	6.41	164.96
	From agricultural (%)	1.58	0.36	0.55	0.1	2.59
SK Bratislava region	From seminatural and natural (km^2^)	2.24	1.18	1.1	1.51	6.03
	From seminatural and natural (%)	0.14	0.07	0.07	0.09	0.37
	From agricultural (km^2^)	15.79	12.83	12.41	5.18	46.21
	From agricultural (%)	0.97	0.78	0.76	0.32	2.83
PL Krakowski subregion	From seminatural and natural (km^2^)	0.17	0.32	0.71	2.45	3.65
	From seminatural and natural (%)	0.004	0.008	0.02	0.06	0.092
	From agricultural (km^2^)	83.01	183.88	8.3	50.82	326.01
	From agricultural (%)	2.05	4.54	0.2	1.26	8.05

Artificial areas increased in time with varying intensities in all the cases. In Pest County and Bratislava Region, the dynamics of the transformation of agricultural areas is reduced now. The peak was in the first period. The ratio declined particularly intensively in the Hungarian region. In the Krakowski subregion, on the other hand, the area of new artificial areas resulting from the transformation of agricultural land was more than twice as large between 2000 and 2006 than from 1990 to 2000. As regards changes in natural and seminatural areas, a slight decrease was identified in Bratislava Region and an upward trend in the Krakowski subregion, while Pest County exhibited a changing trend.

The total loss of natural and seminatural areas in Pest County was 17.14 km^2^, while agricultural land losses amounted to 165 km^2^. The peak was observed between 1990 and 2000, when 100.73 km^2^ of the agricultural area was transformed into artificial surfaces (later only 22.65, 35.17, and 6.41 km^2^). Considering natural and seminatural areas, the peak was also in the first period with 7.01 km^2^. Not much less, 5.62 km^2^ was converted from 2006 to 2012. After 2012, the sprawl slowed down considerably. In Bratislava Region, a 52.24 km^2^ area was developed, from which 6.03 km^2^ came from seminatural and natural areas and 46.2 km^2^ from agricultural land. The peak here was again in the first period after the political changes with 15.79 km^2^ of lost agricultural area. The lowest loss value (5.18 km^2^) was in 2012–2018. In the Krakowski subregion, after an intensively increasing trend between 2000 and 2006, more than 180 km^2^ of artificial areas were created from agriculture. After a slowdown (2006–2012), the trend grew again. In total, almost 330 km^2^ of agricultural, natural, and seminatural areas have been converted into artificial areas since 1990, which is the highest absolute value among the analysed regions.

**Figure 2:**
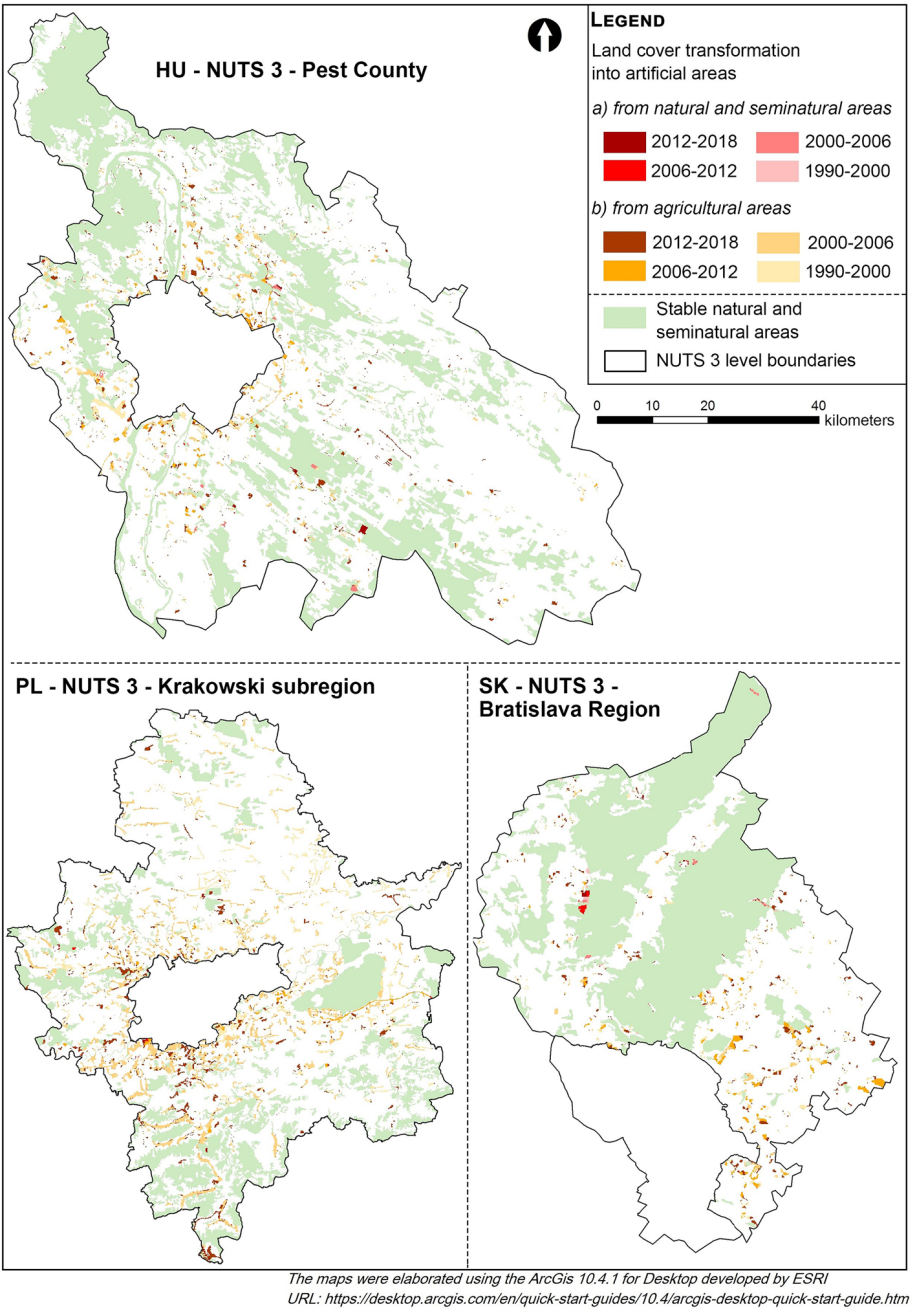
Land cover transformations into artificial areas 1990–2018.

Regarding the spatial location (Fig. 2) of the new built-up areas, most of them exhibit a higher density and concentration around the core city. The concentration is especially intensive around Kraków.

In all the analysed regions we can witness strong suburbanisation around the core city sometimes with different arial focus in the different periods, which is characteristic for all examined regions, with a more dispersed process in Krakow region. A common trend is that main roads and motorways direct the construction process as lines of commuting and cause further wave of urban growth. Large forested areas and mountains such as Carpathian Mountains (Little Carpathians) in the vicinity of Bratislava or Buda mountains in Budapest region put a stop for urban expansion.

#### Does protected green infrastructure remain stable?

**Figure 3:**
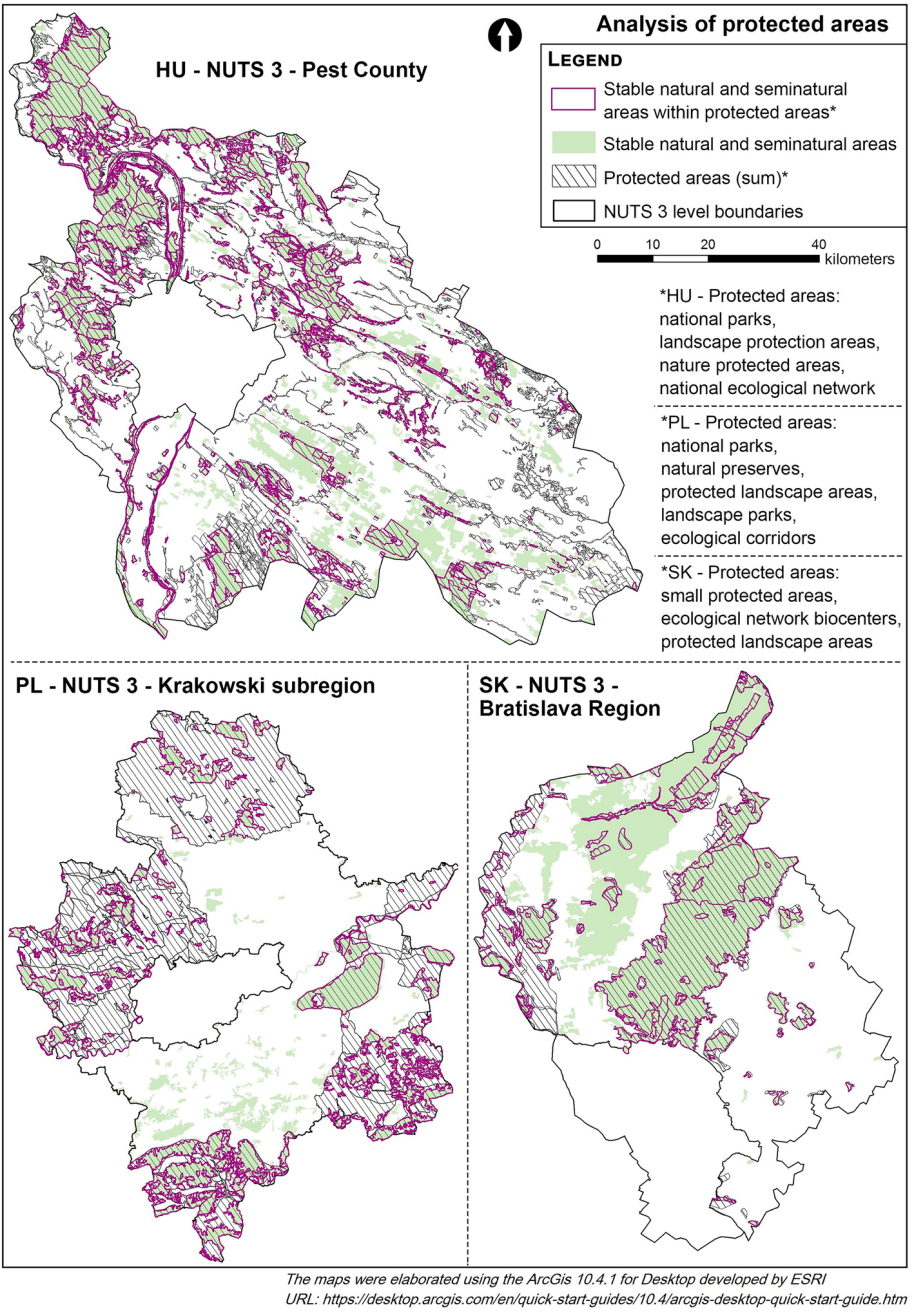
Spatial distribution of stable natural and seminatural areas in relation to protected areas.

Twenty-seven per cent of the analysed Central and Eastern European peri-urban areas were stable natural and seminatural areas (Pest County: 29.0%, Bratislava Region: 42.9%, the Krakowski subregion: 18.5%). It means they were not transformed from 1990 to 2018. In Hungary, the majority of the stable areas are under different levels of protection. Large blocks of forests are located in landscape protection areas (Buda and Gödöllő) and National Park Duna-Ipoly. Large areas of pastures and wetlands remained intact south of Budapest in the plain landscape of Kiskunság National Park (Fig. 3). Bratislava Region has two large stable natural and seminatural areas stretching north from Bratislava (Fig. 3). They were preserved due to varied geographical conditions between the Eastern Alps and the Carpathians, the forested massif of the Little Carpathians, which is under multi-level protection (Natura 2000 site, protected landscape, and ecological network biocenters), and the forested Trnava Uplands. In the Krakowski subregion, stable natural and seminatural areas are dispersed. The largest stable natural area is in the eastern part of the study area (Fig. 3). This area is protected as an ecological corridor and on the international level as a Natura 2000 site. A high concentration of stable areas can also be observed in the southern part of the Krakowski subregion. Importantly, some of this land (closer to Kraków) is not covered by any means of legal protection.

The share of protected areas in the total study areas varied when aggregate areas of all national conservation means are considered (except for Natura 2000 sites as these were designated in 2004 and in most cases they overlap with the national system): more than one-third of the study areas in Hungary and Slovakia is under some kind of nature protection scheme (HU 33.23%, SK 35.61%), while in Poland it is half of the territory of the Krakowski subregion (PL 55.30%) (see Table A2 in Appendix). Natura 2000 sites play an important role only in Pest County, where the share of these areas in the total NUTS 3 area expressed with I_P2000_ is 22.15%. In the case of Bratislava Region, the ratio is 15.41%, while the share of protected landscape area in the area of the NUTS3 reflected by IP_PLA_ is almost 30%. For the Krakowski subregion, the role of this means of protection appears to be the lowest. It is evident from the value of I_P2000_ of merely 4.69%. In the Krakowski subregion, as in the case of Bratislava Region, the largest area is occupied by a protected landscape area (I_PPLA_ = 22.80%).

Considering stable natural and seminatural areas in national-level protected areas (Table A2), the highest values were found in Bratislava Region (73.24%) and Pest County (54.65%). The lowest share was in the Polish region (26.49%). The Krakowski subregion and Bratislava Region have the highest proportion of stable cover in Natura 2000 sites (Slovakia: IP_2000SNS_ = 81.31%, Poland: IP_2000SNS_ = 76.04%). In Hungary, we found rather high stability regarding national-level nature protection areas, especially national parks (IP_NPSNS_ = 84.34%) and landscape protection areas (IP_LPASNS_ = 77.57%).

On the other hand, considering the ratio of protected stable areas compared to total stable natural and seminatural areas in the Slovakian and Hungarian study areas, all protection means seem effective because the share of protected stable areas exceeds 60%. However, this value is even higher in the Krakowski subregion (78.85%). Ecological corridors in the Polish study area account for the largest share of stable natural and seminatural areas (ISNS_PEC_ = 44.68%). Protected landscape areas in Slovakia have the highest share (ISNS_PPLA_ = 49.33%) among all stable protected areas. In Hungary, the values of stable natural areas under the National Ecological Network reflect the difference in the effectiveness of protection (ISNS_PCA_ = 41.55%; ISNS_PEC_ = 14.98%; ISNS_PBF_ = 5.93%). In the Bratislava Region, the value of ISNS_P2000_ shows that 85.88% of stable areas are under Natura 2000 protection. In Pest County, ISNS_P2000_ is 43.50%, while in the Polish study area, ISNS_P2000_ reaches only 19.19.

To sum up the results, nature protection means seem to be an important tool in all regions. However, their effect is limited as their coverage is around one-third of the total area except Poland, where the proportion is larger. Still, the ratio of stable natural and seminatural areas is lower there than in protected areas in the other countries. Furthermore, the effectiveness of different nature protection tools varies. There are three categories of means of protection. First, there are the traditional national-level nature protection types (national parks, landscape protection areas, nature reserves, etc.). Then there are European-level nature protection areas, i.e. Natura 2000 sites, followed by ecological networks, mostly with the lowest level of protection. In all the study areas, Natura 2000 areas cover a large proportion of stable natural and seminatural areas. This value is also high in the Slovakian and Hungarian national-level protected areas, but quite low in the Polish region. Based on this rough assessment and mostly exploring the national trends compared to other countries, nature protection tools in the Polish region cover larger areas but are less capable of stopping land use changes.

## Discussion

### Overlapping the results of the different research methods

In our study, we highlighted the main differences and the effectiveness of spatial policies and green infrastructure protection tools. In case we superimpose and interpret the results of the different research methods, we see interesting correlations. Considering the location and proportion of stable natural and seminatural areas nature protection seems to be a more effective tool in Poland as almost 80% of stable areas are in nature protection areas. This rate is lower in the Hungarian and Slovakian study areas, where however in the not protected, „everyday landscapes” the negative land use changes were not so intense either. The reason for that could be the more effective spatial planning tools especially considering the lower rate of growth of built up areas in the Hungarian and Slovakian capital regions. The case of Bratislava is a little more complex, the lower rate of urban growth can be misleading, as the region is located in border region where an important destination of suburbanisation are Hungarian villages as well. So Hungarian villages absorb and lower urban growth from the Slovakian capital region. So, the land use plan of Budapest agglomeration seems a more effective tool compared to the other cases in spite of its one sidedness with the strong focus on regulation and its late introduction. These results draw the attention on the significance and importance of spatial planning.

### Effectiveness of nature protection tools

A wide range of tools is available to control development. Among them, nature protection seems to be quite effective in preserving ecological values, based on the high proportion of stable natural and seminatural areas in protected land. Ecologically valuable areas and ecological connections are also protected with lower-level protection in the analysed countries. It is evident in Hungary, where a larger buffer zone designation in the framework of the ecological network could perhaps also be a good tool for combating urban sprawl in the agglomeration area. In Slovakia, ecological corridors are not designated as protected areas over their full width. Only connections between biocenters are designated and are rather loosely protected. Further research would be necessary to explore the main characteristics of stable areas from the point of view of land use types. However, nature protection is not suitable tool to control urban growth as we witnessed in Krakowski region the highest rate of protected areas (55%) and highest rate of growth of artificial areas as well. In general, landscape conditions, geography, and forests provide the strongest barrier to new structures and infrastructure, which indicate further development, and the most exposed areas are those around the existing built-up areas and along infrastructure lines.

### Effectiveness of spatial planning tools

After 1990, the former socialist countries without any experience faced strong and intensive suburbanisation^59^. At the same time, they had to transform their socio-economic systems and develop a new spatial policy^60^. Several aspects of spatial policy originate from experiences related to socialism. For example, the weakness of regional-level tools or a low level of bottom-up or intermunicipal cooperation. Our results have shown a high scale of urban expansion in Poland, consistent with other studies^61^. The main problem is that in the absence of local spatial development plans, municipalities enjoy great freedom in regulating the issue of spatial development. However, masterplans must take into account the actual need for new investment areas from 2016^53^, which is yet another measure to counteract urban sprawl. The effectiveness of the masterplans is hindered by the fact that they are not binding on landowners despite their importance in the development processes^62^. Therefore, in the absence of a local plan, urban sprawl is basically unregulated. Reacting to the high scale of urban sprawl, Poland is preparing a spatial planning reform with the main objective to prevent the dispersion of development into agricultural, forest, and naturally valuable areas.

Urban sprawl in Bratislava Region is a result of the economic attractiveness of the capital city and broader macro-regional situation (proximity to Austria, Hungary, and the Czech Republic and their major cities Vienna, Brno, and Győr). Migration from the east of the country is strong even today. The decentralised planning system could not effectively regulate this. Municipalities were usually welcoming new developments and new citizens as harbingers of increased economic activity. However, the actual results of urban sprawl cannot really be investigated by analysing the study area due to the border situation^37^. Over the last 10 years, Slovak citizens grew interested in buying property in Austrian and Hungarian cross-border areas as a result of increasing real estate prices in Bratislava and its surroundings. Hence Bratislava is overgrowing its administrative borders and urban sprawl is partly counterbalanced by neighbouring cross-border regions^63^.

The results show that other tools are also necessary and it is mostly the frameworks and regulatory tools of spatial planning that can protect these ‘exposed’ landscapes in peri-urban areas. The national level spatial plans are not focused enough to deal with such specific areas extremely exposed with urban expansion. Local-level administration has the strongest spatial planning competences related to space management. From the point of view of combating urban sprawl, considering rational land management in agglomeration areas, the capabilities of municipalities are limited, making regional-level tools necessary. Other researchers highlighted the need for stronger supra-municipal coordination to foster a more rational land use policy by avoiding competition between local authorities and preventing oversized designated building zones^64^. In terms of spatial planning tools, the analysed countries lack focused, regional-level tools. Hungary, Slovakia, and Poland have decentralised systems. Mostly just the main guidelines and framework rules are set on the national level. On the regional level, there is a certain degree of spatial-planning autonomy, however quite weak. Hungary has the most centralized system, so the top-down regulatory approach is the strongest in Hungary. The effectiveness of regional-level spatial tools in combating urban expansion is hindered by the weakness on the regional level or the spatial differences between administrative and functional areas/agglomeration areas (PL, HU). However, a specific spatial planning tool was defined for the Budapest agglomeration zone. Its impact was somewhat damped because there was no moratorium for designating new development areas before it was enacted. As a result, most of the local municipalities used their last chance and now there are vast areas buildable areas in the vicinity of Budapest. The land use plan had also questionable tools such as preventing the merging of urban areas as discontinuous urban fabric has less "sprawling character" than continuous urban fabric see "leapfrog development"^65^. Considering the growth rate in the different periods the plan was launched quite late in 2005 as after 2000 there was a lower level of growth, and it couldn’t prevent the new wave after 2006. Furthermore, the agglomeration zone does not serve as a strategic regional cooperation platform, so the spatial planning system is ‘one-sided’; a strategic approach is missing. Centralized spatial planning system can offer stronger regulation policy however due to the lack of focused or misplaces tools this policy can have deficiencies on the long run. The effectiveness of long term, regional level planning could be enhanced by setting up an intermunicipal cooperation between local communities affected by the suburbanisation process and between the capital. Unfortunately, the continuous centralization process present in Hungary doesn’t provide suitable, favourable circumstances for such bottom-up initiative.

As mostly the national level tools (such as national level spatial plan, nature protection tools) are not effective enough, regional level tools or institutions should be enhanced. Regional governance could be an important platform for harmonising development needs. There are several examples in Western Europe, where a bottom-up approach fosters the cooperation of stakeholders. On the contrary, regional cooperation platforms are glaringly missing in Eastern and Central European countries. In Germany, many legal forms of intermunicipal cooperation are available. The public recognized that—owing to strong interdependencies within urban regions—it is important to move from isolated actions to an overall, regional approach, even though it often restricts the autonomy of local authorities^66^. The case of Munich region could be a good example for Budapest agglomeration as well, where the Functional Urban Area of Munich is equal to the Regional Planning Association Munich (RPV), which is the legally planned association of municipalities. France has a specific situation, where the highly fragmented local governmental system has been fuelling intermunicipal cooperation for centuries^33,67^. For example, there is able strategic planning for the metropolis region in Lille, Lyon, and Rennes there^68^. Nilsson and Nielsen^69^ also pointed out the need for stronger regional-level competencies in land use planning and emphasized that the EU can promote a more integrated urban–rural development with appropriate targeted funding. The EU should encourage the development of national policies and frameworks for metropolitan areas through:

- setting chief priorities for regional development in partnership agreements with member states;
- offering new instruments to encourage metropolitan cooperation in urban development programmes, such as the Integrated Territorial Investments (based on Article 36 of Common Provision Regulation, REGULATION (EU) No. 1303/2013) programme, under which successful initiatives were launched in the Czech Republic and Poland;
- using existing European territorial cooperation programmes (URBACT, INTERREG, ESPON) to highlight the importance of functional urban areas related to sustainable spatial planning^68^.

There are several possibilities to ensure a more balanced spatial development in metropolitan regions in Central and Eastern European countries:

- An effective tool to fight the challenges agglomerations face could be to set up a metropolis-level institution with a broad array of functions and a strong strategic planning authority. Strengthened metropolitan planning may assist in forming more compact communities and supporting more environmentally friendly modes of transportation by coordinating development and land use. By coordinating development, mitigating negative impacts, and capitalising on opportunities, metropolitan planning can help to create more livable, sustainable, and prosperous metropolitan areas.
- Another option is to develop a framework/platform based on voluntary cooperation. Cities should take a more proactive approach towards their agglomeration areas, acting in a bottom-up manner to create a more coordinated development model.

Planning for functional urban areas should be prioritised in the EU Cohesion Policy and use of EU funds. In 2014, local communities in the Kraków FUA formed a partnership, the Kraków Metropolis Association (KMA), to develop and implement the ITI strategy. The experience to date shows that this cooperation can have added value for the metropolitan area^70^. Such cooperation should be expanded and even institutionalised in regional development programmes^68^. In Slovakia, there is a strong urge to strengthen the territorial approach. Cooperation platforms were created to implement integrated territorial investments for 2021–2027: one at the level of NUTS3 and one at the level of functional urban regions such as the Bratislava functional urban area. Still, the proposed implementation model has a very limited integrative approach because of the strong centralisation of project approval procedures at the national level. Hungary has a simplified version of the EU initiative with no actual cooperation among cities.

Regarding the dynamics of the growth in artificial areas the first period, right after the political changes, is the most intensive in Pest County and Bratislava Region. In contrast, the most significant changes in the Polish study area happened in the period between 2000 and 2006. The difference may be explained by the capital city region status in the first two countries^71^. After the fall of socialism, private green field investments focused in the central region, and suburbanisation was the most intensive in the surroundings of the capital. The Polish study area did not include an area close to a capital city, only the Krakowski sub-region. Hence, its development came at a later stage.

Parallel to our research, several studies highlighted the threat to farmland, even though all the countries enacted specific regulations to protect agricultural land. The pressure around large cities is high^49,72,73^. Development scenarios usually predict further and continuous development, especially along the borders of urban areas, and further loss of agricultural areas^8^ highlighting the need for other tools to protect agricultural areas in peri-urban zones. However, it is a strategic objective in all the countries. The greenbelt can be a really complex, and yet flexible, tool for protecting unbuilt peri-urban areas. However, none of the countries applied this kind of tool.

Our results show that the investigated Central and Eastern European countries do not really make use of the wide range of informal, bottom-up possibilities, such as intergovernmental cooperation or other tools. Regional-level spatial policy tools are mostly weak and are not capable of controlling sprawl. More focused spatial tools would be necessary with stronger control over unbuilt areas. Furthermore, strengthening the participative, cooperation-oriented approach would be important in the future^74^. As regards economic tools, Polish cities offer incentives for greening the city, which nevertheless fail to foster a compact settlement structure. A specific incentive for promoting brownfield investments exists just in Hungary, but it being a newly introduced tool, there is still no data regarding its effectiveness. Regardless of any regulatory approaches, better use of economic incentives and informal tools would be useful to foster rational land management in peri-urban areas of Central and Eastern European countries in the future.

Environmental Impact Assessment (Directive 2014/52/EU) and/or Strategic Environmental Assessment (Directive 2001/42/EC) guidelines of the European Union applicable in all three countries state the need to formulate compensation measures for projects/strategic documents and prescribe the integration of ecological values into strategic documents and assessment. Still, most gaps and problems occur during implementation and economic interests often prevail in practice^33^.

## Conclusions

Based on our research comprising parallel spatial and policy analyses, we can conclude the following key insights concerning the level and growth trends for artificial areas, loss of green infrastructure, and policy tools for protecting green infrastructure and combating urban sprawl:

1. After decades of socialism, a very intensive urban sprawl process started in the now democratic countries. It mostly slowed down after 2000 but with varying trends. The capital regions (HU and SK) had a peak after 1990 and the Polish study area between 2000 and 2006.
2. The most remarkable characteristic of the changes was that 88–98% of the new artificial areas came from agricultural land.
3. Almost one-third of the area of the analysed Central and Eastern European peri-urban areas (with quite large differences by country) were stable natural and seminatural areas, which were not transformed from 1990 to 2018.
4. The relation between the stable natural and seminatural areas and nature protection means, such as national parks, demonstrated that traditional nature protection tools are effective in preserving natural and seminatural areas.
5. Stable areas not located close to the core city are in most cases, so the protection of landscapes exposed to urban sprawl needs specific tools.
6. Regarding spatial planning, the local level has the most authority over decisions about spatial development. The competencies of the regional level are weak or the authorities' jurisdictions do not overlap with the regions most exposed to urban sprawl.

To summarise the instruments and tools for green infrastructure protection, the analysed countries do not have specific tools for controlling urban sprawl, except for the Budapest agglomeration. The regulatory approach is strong, and the bottom-up incentives such as fostering inter-municipal cooperation are absent in the analysed countries. There are no greenbelts around the urban areas. Nature conservation as a traditional tool for protecting ecological values is important in all the analysed regions. We did not find other tools, such as economic or other incentives, for fostering a compact settlement structure. Just some Polish cities had initiatives for developing and protecting green infrastructure.

### Supplementary Information

Supplementary Information 1.

Supplementary Information 2.

Supplementary Information 3.

## Data Availability

The datasets used and/or analysed during the current study available from the corresponding author on reasonable request.
