# Supplementary material for: Policy instruments as a trigger for urban sprawl deceleration: monitoring the stability and transformations of green areas
Source: Sci Rep. 2024 Feb 1;14:2666. doi: 10.1038/s41598-024-52637-9 (PMC10834971; doi:10.1038/s41598-024-52637-9)
Supplement: Supplementary file 1 — Supplementary Information 1. [file 41598_2024_52637_MOESM1_ESM.docx]

**Appendix A1.** A universal questionnaire in the field of regulatory, economic, and informational and motivation tools for controlling urban sprawl and protecting green infrastructure

| **Group** | **Subgroup** | **Questions** |
| --- | --- | --- |
| **Spatial planning** | tools in the general spatial planning framework | What are the levels of spatial planning in the NUTS 3? (R) |
|  |  | What are the tools for regional planning generally? (R) |
|  |  | What are the strategic planning tools in the region? How do they reflect on urban sprawl? (R) |
|  |  | Is there any planning association/institution, or inter-municipal cooperation which is relevant? (IM) |
|  |  | Are any activities being undertaken to raise public awareness of spatial planning? (IM) |
|  | special actions to combat urban sprawl | What kind of regulatory tools exist in the region for controlling urban sprawl? (R) |
|  |  | Are there any special regulations for the protection of unbuilt areas? (R, E) |
|  | green infrastructure planning and protection tools | What kind of relevant green infrastructure planning tools exist in the NUTS 3? (R) |
|  |  | Do these plans contain regulations for ecological networks? If yes, what are they? (R) |
|  |  | What are the most relevant nature protection tools in the NUTS 3? (R) |
|  |  | Are there any tools for compensation for the loss of ecological values due to development? (E) |
| **Legal nature protection means** | differences in levels of protection | What are the most relevant nature protection institutions in the NUTS 3? (R) |
|  |  | What means of national-level nature conservation are found in NUTS 3? (R) |
|  |  | What means of international-level nature conservation are found in NUTS 3? (R) |
|  | tools for the protection of green infrastructure, ecological network/corridors, and greenway planning as a specific tool | What kind of relevant green network planning or protection tools exist in the region? (R) |
|  |  | What are the rules for them? (R) |
| **Economic instruments** | specific incentives | Are there any specific incentives for fostering a compact settlement structure? (E) |
|  |  | Are there any specific incentives for fostering green infrastructure protection? (E) |
|  |  | Are there any specific incentives to take into account the need to pay additional costs for the increase in property value? (E) |

Note: (R) – regulatory instruments, (E) – economic instruments, (IM) – informational and motivational instruments.
