# Supplementary material for: Policy instruments as a trigger for urban sprawl deceleration: monitoring the stability and transformations of green areas
Source: Sci Rep. 2024 Feb 1;14:2666. doi: 10.1038/s41598-024-52637-9 (PMC10834971; doi:10.1038/s41598-024-52637-9)
Supplement: Supplementary file 2 — Supplementary Information 2. [file 41598_2024_52637_MOESM2_ESM.docx]

**Appendix A2.** Structure of nature protection means and their share in stable natural and seminatural areas

| NUTS 3 | **Name of protection means** | **Area** | **Proportion to the total area of NUTS 3 region** | **Stable natural, seminatural area within the given (x) protection means** | | |
| --- | --- | --- | --- | --- | --- | --- |
|  |  |  |  | **Area** | **Proportion to the total area of the given (x) protection means** | **Proportion to total area of stable natural and seminatural areas** |
|  |  |  | I_Px_ |  | IP_xSNS_ | ISNS_Px_ |
|  |  | [km^2^] | [%] | [km^2^] | [%] | [%] |
| **HU-Pest County** | Natura 2000 | 1,412.45 | 22.15 | 805.28 | 57.01 | 43.50 |
|  | National park | 466.07 | 7.31 | 393.09 | 84.34 | 21.24 |
|  | Landscape protection area | 270.07 | 4.23 | 209.49 | 77.57 | 11.32 |
|  | Nature protection area | 7.34 | 0.12 | 3.23 | 44.01 | 0.17 |
|  | NEN-ecological corridor | 728.14 | 11.42 | 277.22 | 38.07 | 14.98 |
|  | NEN-core area | 1,107.17 | 17.36 | 769.21 | 69.48 | 41.55 |
|  | NEN-buffer zone | 275.12 | 4.31 | 109.68 | 39.87 | 5.93 |
|  | Ramsar locations | 68.50 | 1.07 | 51.06 | 75.54 | 2.76 |
|  | ***Protected areas: NP, LPA, NPA, NEN** | **2,119.63** | **33.23** | **1,158.32** | **54.65** | **62.58** |
| **SK-Bratislava Region** | Natura 2000 | 855.88 | 15.41 | 602.40 | 81.31 | 85.88 |
|  | Small protected area | 93.30 | 5.71 | 70.23 | 75.28 | 10.01 |
|  | Ecological network biocenters | 222.30 | 13.60 | 172.09 | 77.41 | 24.53 |
|  | Protected landscape area | 477.34 | 29.20 | 346.45 | 72.58 | 49.33 |
|  | Ramsar locations | 167.47 | 10.24 | 75.76 | 45.24 | 10.80 |
|  | ***Protected areas: SMA, ENB, PLA** | **583.24** | **35.61** | **427.14** | **73.24** | **60.90** |
| **PL-Krakowski subregion** | Natura 2000 | 190.04 | 4.69 | 144.51 | 76.04 | 19.19 |
|  | Nature and landscape complexes | 50.40 | 1.24 | 3.12 | 6.19 | 0.41 |
|  | Natural preserves | 72.51 | 1.79 | 40.489 | 55.83 | 5.38 |
|  | Protected landscape area | 924.53 | 22.80 | 167.81 | 18.15 | 22.28 |
|  | National parks | 86.63 | 2.14 | 18.82 | 21.72 | 2.50 |
|  | Ecological corridors | 595.16 | 14.68 | 336.49 | 56.54 | 44.68 |
|  | Landscape parks | 840.72 | 20.74 | 157.66 | 18.75 | 20.93 |
|  | *****Protected areas: NPR, PLA, NP, EC, LP** | **2,241.90** | 55.30 | **593.87** | 26.49 | **78.85** |

Labels of protection means (x): 2000 – Natura 2000; NP – national park; LPA – landscape protection areas; NPA – nature protection areas; SMA – small protected area; ENB – ecological network biocentres; PLA- protected landscape area; RL – Ramsar locations; NPR – natural reserves; EC – ecological corridors; LP – landscape parks; CA - NEN-core area; BZ - NEN-buffer zone; NEC - NEN-ecological corridor
