# Supplementary material for: Policy instruments as a trigger for urban sprawl deceleration: monitoring the stability and transformations of green areas
Source: Sci Rep. 2024 Feb 1;14:2666. doi: 10.1038/s41598-024-52637-9 (PMC10834971; doi:10.1038/s41598-024-52637-9)
Supplement: Supplementary file 3 — Supplementary Information 3. [file 41598_2024_52637_MOESM3_ESM.docx]

**Appendix A3.** Land cover categories used in the analyses, based on Corine Land Cover nomenclature

| **Artificial areas:** |
| --- |
| 1.1 Urban fabric (1.1.1., 1.1.2.) |
| 1.2 Industrial, commercial and transport units (1.2.1, 1.2.2, 1.2.3, 1.2.4) |
| 1.3 Mine, dump and construction sites (1.3.1, 1.3.2, 1.3.3) |
| 1.4 Artificial, non-agricultural vegetated areas (1.4.1, 1.4.2) |
| **Agricultural areas:** |
| 2.1 Arable land (2.1.1, 2.1.2, 2.1.3) |
| 2.2 Permanent crops (2.2.1, 2.2.2, 2.2.3) |
| 2.3 Pastures (2.3.1) |
| 2.4 Heterogeneous agricultural areas (2.4.1, 2.4.2, 2.4.3, 2.4.4) |
| **Natural and seminatural areas:** |
| 3.1 Forests (3.1.1, 3.1.2, 3.1.3) |
| 3.2 Shrub and/or herbaceous vegetation associations (3.2.1, 3.2.2, 3.2.3, 3.2.4) |
| 3.3 Open spaces with little or no vegetation (3.3.1, 3.3.2, 3.3.3, 3.3.4, 3.3.5) |
| 4.1 Inland wetlands (4.1.1, 4.1.2) |
| 4.2 Coastal wetlands (4.2.1, 4.2.2, 4.2.3) |
| 5.1 Inland waters (5.1.1, 5.1.2) |
| 5.2 Marine waters (5.2.1, 5.2.2, 5.2.3) |

Source: Own study based on Corine Land Cover
